# Conserved BK Channel-Protein Interactions Reveal Signals Relevant to Cell Death and Survival

**DOI:** 10.1371/journal.pone.0028532

**Published:** 2011-12-09

**Authors:** Bernd Sokolowski, Sandra Orchard, Margaret Harvey, Settu Sridhar, Yoshihisa Sakai

**Affiliations:** 1 Otology Laboratory, Department of Otolaryngology – Head and Neck Surgery, University of South Florida, College of Medicine, Tampa, Florida, United States of America; 2 European Molecular Biology Laboratory, European Bioinformatics Institute, Wellcome Trust Genome Campus, Cambridge, United Kingdom; 3 Department of Informatics, University of Bergen, Bergen, Norway; University of Houston, United States of America

## Abstract

The large-conductance Ca^2+^-activated K^+^ (BK) channel and its β-subunit underlie tuning in non-mammalian sensory or hair cells, whereas in mammals its function is less clear. To gain insights into species differences and to reveal putative BK functions, we undertook a systems analysis of BK and BK-Associated Proteins (BKAPS) in the chicken cochlea and compared these results to other species. We identified 110 putative partners from cytoplasmic and membrane/cytoskeletal fractions, using a combination of coimmunoprecipitation, 2-D gel, and LC-MS/MS. Partners included 14-3-3γ, valosin-containing protein (VCP), stathmin (STMN), cortactin (CTTN), and prohibitin (PHB), of which 16 partners were verified by reciprocal coimmunoprecipitation. Bioinformatics revealed binary partners, the resultant interactome, subcellular localization, and cellular processes. The interactome contained 193 proteins involved in 190 binary interactions in subcellular compartments such as the ER, mitochondria, and nucleus. Comparisons with mice showed shared hub proteins that included N-methyl-D-aspartate receptor (NMDAR) and ATP-synthase. Ortholog analyses across six species revealed conserved interactions involving apoptosis, Ca^2+^ binding, and trafficking, in chicks, mice, and humans. Functional studies using recombinant BK and RNAi in a heterologous expression system revealed that proteins important to cell death/survival, such as annexinA5, γ-actin, lamin, superoxide dismutase, and VCP, caused a decrease in BK expression. This revelation led to an examination of specific kinases and their effectors relevant to cell viability. Sequence analyses of the BK C-terminus across 10 species showed putative binding sites for 14-3-3, RAC-α serine/threonine-protein kinase 1 (Akt), glycogen synthase kinase-3β (GSK3β) and phosphoinositide-dependent kinase-1 (PDK1). Knockdown of 14-3-3 and Akt caused an increase in BK expression, whereas silencing of GSK3β and PDK1 had the opposite effect. This comparative systems approach suggests conservation in BK function across different species in addition to novel functions that may include the initiation of signals relevant to cell death/survival.

## Introduction

BK channels are involved in a diversity of physiological processes such as metabolism, signaling, phosphorylation, regulation of neurotransmitter release, and modulation of smooth muscle contractions (reviews in [1]). They are activated by the cooperative effects of two distinct stimuli, membrane depolarization and the elevation in concentration of free cytoplasmic Ca^2+^. The channels assemble as tetramers of pore-forming α-subunits, with the enclosing transmembrane topology (S1, S2, S3, S4) responsible for sensing voltage changes and the pore forming loop structure (S5, S6) conducting K^+^ ions [2]. In addition to the transmembrane domains, the BKα subunit has an extensive cytoplasmic C-terminus (S7, S8, S9, S10), containing many phosphorylation sites [3], [4], two K^+^ conducting regulator (RCK1 and RCK2) domains, a string of aspartate residues known as the Ca^2+^ bowl, and leucine zipper, heme, and caveolin binding motifs (reviews in [5]).

The molecular mechanisms that regulate BK channel behavior in the cochlea remain unclear. In the mammalian cochlea, BK channels are localized basally in synaptic zones of inner (IHC) and outer hair cells (OHC) and extrasynaptic zones located near the apical portion of IHCs [6]. In non-mammals, BK channels are found in close proximity to voltage-gated Ca^2+^ channels, where they facilitate frequency tuning [7]. BK channels are involved in noise-induced hearing loss [8], potentially through activation of ROS pathways by the BK channel and associated proteins like SOD, glutathione peroxidase, and GSTμ [9]. The past decade has revealed an unexpected number of protein-protein interactions that basically modify our view of the localization and functional association of previously identified intracellular proteins. The functional interactions of BK channels with their associated proteins are no exception. The pore-forming and C-terminus domains of BK contain several protein kinase and phosphatase binding motifs that associate with a number of partners to regulate channel gating and signaling pathways. These effectors include cAMP-dependent protein kinase A (PKA), c-Src, proline-rich tyrosine kinase 2 [5], cGMP-dependent PKG [10], PKC [11], and Ca^2+^-dependent phospholipid-binding protein [12]. Moreover, the BK leucine zipper region serves as an anchor for the regulation of BK by a PKA-associated complex [5].

To define and compare BK cellular functions, we performed a highly sensitive mass spectrometry analysis of BK complexes isolated from chick cochlea, followed by validation using reciprocal coimmunoprecipitations (coIPs), bioinformatics, and BK/BKAP functional associations using partial knockdown by siRNAs. Conservation of chick BKAPs was phylogenetically compared with mouse [9] and other vertebrate and non-vertebrate data, by using interactome and ortholog analyses. Major hubs in the BK interactome suggested novel insights into BK-partners, pathways, and cellular processes. Here, we report that there is some overlap in BKAPs found in mouse and chicken. Moreover, BK is involved with proteins and pathways of a survival/apoptotic nature. Some of these proteins are conserved phylogenetically and likely serve a purpose related to the mediation of cell death and/or survival.

## Materials and Methods

### Ethics Statement

Experiments described herein were approved by the University of South Florida Institutional Animal Care and Use Committee (Protocols 3931R, 3482R) as set forth under the guidelines of the National Institutes of Health.

### Coimmunoprecipitation and 2-D gels

A total of 12 cochleae were excised from 15 day-old chicks (*Gallus gallus*) and sonicated in lysis buffer supplemented with protease/phosphatase inhibitors, as described previously [9]. Isolation of the membrane/cytoskeletal fraction was accomplished by centrifuging the lysate at 100 k×*g* for 1 h at 4°C, removing the cytosolic fraction, and solubilizing the pellet with 1% ASB-14 (Calbiochem), which proved to be the most effective in isolating membrane proteins [9]. Fractions were pre-cleared briefly with protein G beads (Invitrogen) and each fraction was set aside for analyses of the total and BK-immunoprecipitated proteomes. Controls for the latter included the use of an irrelevant antibody of the same type (polyclonal antibody to vesicular stomatitis virus, Bethyl Laboratories, Montgomery, TX) and unbound beads. Immunoprecipitation using a polyclonal antibody to BKα (Chemicon, Temecula, CA) was performed using the immunocomplex-capture method, as described previously [9]. Briefly, after capture, beads were washed several times and complexes eluted for subsequent isoelectric focusing (IEF) using pH 3-10 immobilized pH-gradient (IPG) strips (Bio-Rad, Hercules, CA). Proteins were fractionated in the second dimension by SDS-PAGE with 12% gels, Coomassie-stained, and molecular weights approximated using Precision Plus (Bio-Rad) as the protein standard. Spot sets were processed, compared and selected for analysis as described previously [9]. Results from at least two of four replicate experiments were sent for MS analysis.

### Mass Spectrometry

Tandem mass spectrometry sequencing was achieved using an Agilent Technologies (Santa Clara, CA) 1100 liquid chromatograph coupled to an HCT Ultra mass spectrometer (Moffitt Proteomics Facility, Moffitt Cancer and Research Center, Tampa, FL), as described previously [9]. Briefly, peptides were captured and analyzed using 5 µm SB-Zorbax C-18-packed columns on an Agilent Protein ID Chip (G4240-62002). Samples were loaded at the rate of 4 µl/min followed by LC-MS/MS at 300 ηl/min. Five spectra were acquired for each scan and prior precursors were excluded for 60 s.

DataAnalysis software (v. 3.4 Bruker Daltonic GmBH, Bremen, Germany) was used to generate peaklists. Sequences were assigned using the MASCOT search engine (v.2.1.03, Matrix Science, Boston, MA) against the National Center for Biotechnology Information non-redundant database (NCBInr 2006.12.05) selected for bony vertebrates (276,256 entries). The details included accession number, MASCOT score, number of peptides matched, molecular weight, sequence coverage, e-value, delta, score, rank, charge, number of missed cleavages, p value, and peptide sequences. Precursor mass tolerance was ±2.5 Da (monoisotopic) and fragment ion tolerance was ±0.8 Da (monoisotopic). No fixed modifications were selected and variable modifications consisted of carbamidomethylation (C), carboxymethylation (C) and oxidation (M). A maximum of two missed tryptic cleavages was allowed. Peptide assignments were manually verified by inspection of the tandem mass spectra and consistency with expected gas phase fragmentation patterns. Scaffold (v. 01 07 00, Proteome Software, Portland, OR) was used to validate MS/MS peptides and for protein identification (ID). A 95% confidence level was assigned for the score values of individual spectra and peptides were selected as specified by the Peptide Prophet algorithm. In addition, a false discovery rate was determined for the obtained spectra by sampling the file against a reversed database of bony vertebrates using Scaffold for the analysis.

### Database Analyses

The search for additional proteins (i.e., secondary) that interact with primary BKAPs was performed using the Envision tool (www.ebi.ac.uk/enfin-srv/envision) to search the molecular interaction database IntAct (www.ebi.ac.uk) [13], a central repository of manually curated literature data generated by many different experimental techniques. Colocalization data (e.g., cosedimentation) were not included in the final results. Interaction networks were visualized, modeled, and analyzed using Cytoscape (www.cytoscape.org) [14]. Proteins in the network were labeled according to UniProtKB nomenclature and color-coded according to the fraction from which they were obtained (membrane/cytoskeleton vs. cytoplasmic) as well as the database (i.e., IntAct) and subcellular localization.

### Clusters of Orthologous Groups Analyses

Primary and secondary BKAP interactions, for both chicken and our recently published mouse data [9], were analyzed for their conservation of interacting Clusters of Orthologous Groups (iCOGs) across six eukaryotic species including, *E. cuniculi*, *S. cerevisiae*, *A. thaliana*, *C. elegans*, *D. melanogaster*, and *H. sapiens*. Protein IDs in UniProt nomenclature were converted to their corresponding euKaryotic Orthologous Group ID (KOG_ID) by searching the STRING (string-db.org/) database. If either or both proteins of a pair were not identifiable by a KOG, they were removed from the list. Each KOG_ID, of an interacting protein pair, was used to search the NCBI KOG protein database, for a corresponding KOG cluster [15] using BLASTO [16]. The database was searched for the presence/absence of each KOG_ID among the aforementioned six species and a matrix table generated to determine conservation. Values were assigned as follows: 0 if neither of the pair was found, 0.5 if one of the pair was found, and 1 if both members were present. Scores for all six species were used as input to generate three dendrograms (chick, mouse, chick/mouse interologs) using R language to geneate a heatmap (http://www2.warwick.ac.uk/fac/sci/moac/students/peter_cock/r/heatmap).

### Reciprocal Coimmunoprecipitation

This procedure was accomplished using chick cochleae as described previously [17], with the appropriate antibody (Table S1) to 14-3-3γ, annexin A5 (Anxa5), CTTN1, glucose-related protein 78 (GRP78), hippocalcin1 (Hpcal1), heat shock protein (HSP) 60, 70, and 90, lamin A (LMNA), lin-7 homolog C (Lin7c), PHB, ras-proximate 1 (RAP1), synaptosomal-associated protein 25 (SNAP25), superoxide dismutase 1 (SOD1), VCP, and STMN. Briefly, 2.5–6 µg of antibody was used in the immunocomplex-capture technique. Immunocomplexes were eluted from protein G beads by heating at 70°C for 10 min in sample buffer (Sigma-Aldrich, St. Louis, MO). Following fractionation on 10% SDS-PAGE gels and transfer to nitrocellulose membrane (Protran BA; Schleicher & Schuell, Keene, NH), blots were probed with an anti-BKα polyclonal antibody (Chemicon) at 1∶275, followed by donkey anti-rabbit HRP-conjugated secondary antibody at 1∶5000 (Amersham, Piscataway, NJ). The positive control consisted of immunoprecipitating BK using 6 µg of anti-BK antibody, whereas negative controls included preadsorption of antibody with antigen and uncomplexed beads. Immunoreactive bands were developed using enhanced chemiluminescence (ECL; Amersham) and Magic Mark XP (Invitrogen, Carlsbad, CA) was used as the protein standard to estimate relative mobilities.

Six µg of a polyclonal anti-BK antibody (Chemicon) was used to coIP Akt1, GSK3β, or PDK1. To probe for Akt1, a western blot was performed with a rabbit monoclonal anti-Akt1 antibody at 1∶1000 followed by a mouse anti-rabbit secondary antibody (Cell Signaling Technology, Beverly, MA) at 1∶2000. GSK3β immunoblotting was performed with an anti-GSK3β antibody at 1∶1000 followed by a monoclonal anti-rabbit light-chain-specific secondary antibody (Jackson ImmunoResearch, West Grove, PA) at 1∶5000. PDK1 immunoblotting was performed with an anti-PDK1 antibody at 1∶750 followed by the same secondary used for GSK3β at 1∶5000. Additionally, each of these three proteins was immunoprecipitated for comparison to the coIP using BK. To immunoprecipitate each protein Akt1, GSK3β, and PDK1 were used in amounts of 0.52, 12, and 5 µg, respectively, followed by a western blot as described above. The same amounts were used for a reciprocal coIP of BK followed by immunoblotting with an anti-BK antibody at 1∶150 followed by a donkey anti-rabbit HRP-conjugated secondary antibody at 1∶5000 (Amersham).

### HA-tagging of a BKα Splice Variant from Chick Cochlea

A tandem hemagglutinin- (HA) tagged cSlo1 (α-subunit, DEC type, Acc. No. U23821) vector was constructed using pcDNA3.1-cSlo1 as a PCR template. The forward primer, 5′-CGCGGATCCACCATGGGAT ACCCTTACGACGTTCCTGATTACGCTTACCCTTACGACGTTCCTGATTACGCTATGAGTAACAATATCAACGCCAA-3′, included a *Bam*HI (5′) site and a tandem HA-tagged sequence for linkage to the N-terminus of BK-DEC. The reverse primer, 5′- CGCGGATCCCCTGAGTTC TCCACCAAATGT-3′, was set for the cSlo1 *Bam*HI site. PCR (MJ Thermocycler, Bio-Rad) was accomplished using Taq DNA polymerase (Invitrogen) under the following conditions: 94°C for 2 min, 35 cycles at 94°C for 30 sec, 55°C for 30 sec, 68°C for 2 min, and a final cycle at 72°C for 10 min. The PCR product was purified (PCR Purification Kit, QIAGEN, Valencia, CA) and cut with *Bam*HI (New England Biolabs, Ipswich, MA) at 37°C overnight. This fragment was gel-purified (StrataPrep DNA gel extraction kit, Stratagene, Santa Clara, CA) and ligated to the *Bam*HI site of pcDNA3.1-cSlo1 using T4 ligase (Roche, Indianapolis, IN). All primers were acquired from Integrated DNA Technologies (San Diego, CA).

### BKAP siRNA Studies

Prior to initiating these experiments, a manual review of the literature showed that partial knockdown of these RNAs was not lethal to the cell. RNAi experiments used custom-designed siRNAs to target endogenous 14-3-3γ, Anxa5, γ-actin, LMNA, VCP, SOD, Akt1, PDK1, and GSK3β (Stealth Select RNAi, Invitrogen). Negative controls consisted of scrambled RNAs (scRNA) for low-, medium-, and high-GC content RNAi. Transfections were performed using 8 µl/plate of Lipofectamine2000 (Invitrogen), 1 µg of HA-tagged *cSlo*/pcDNA 3.1 and pooled siRNAs of 300 nM 14-3-3γ, Akt1, Anxa5, γ-actin, and GSK3β, or 450 nM LMNA, SOD1, VCP, and PDK1. SiRNAs were mixed in a 1∶1∶1 ratio targeted to the sense strands (5′-3′, Table S1). scRNAs were transfected in a similar manner using equal concentrations. Cells were harvested and processed for electrophoresis and immunoblotting as described previously [9].

## Results

### Proteomic analysis of BKAPs and reciprocal coIP verification

**Figure 1:**
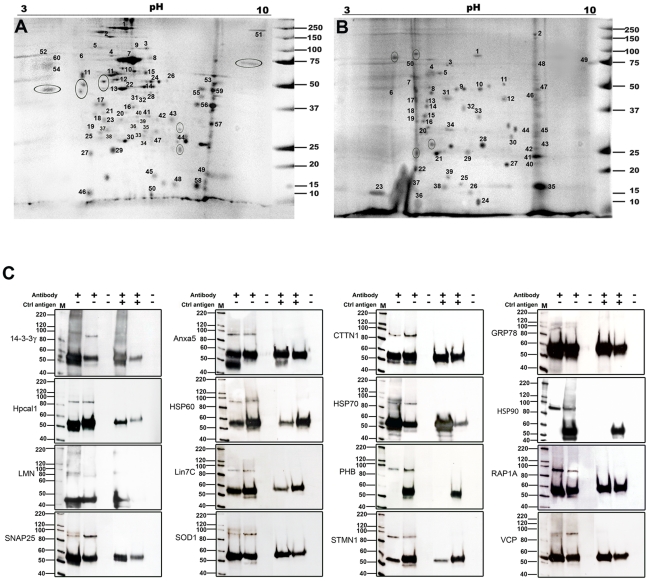
BK interactions resolved by two-D gel electrophoresis and reciprocal coIP. (**A,B**) Chick membrane/cytoskeletal and cytoplasmic fractions show 60 and 50 distinct numbered spots from the immunoprecipitated gels that were subjected to LC-MS/MS analysis. Regions delimited by ovals represent proteins common to both fractions. (**C**), Sixteen representative examples of BKAP reciprocal coIPs (lane 2; +, −) and BK IPs (lane 3; +,−) reveal immunoreactive peptide species of ∼90 or 120 kDa for BK in chick cochlea. The negative control in which anti-BK antibody was preadsorbed with peptide, did not produce immunoreactive bands for either the BKAP reciprocal coIPs (lane 5; +,+) or the BK IPs (lane 6; +,+). The 55 kDa bands correspond to heavy immunoglobulin (IgG), resulting from cross-reactivity or the use of antibodies of the same clonality. Bead controls consisted of lysate mixed with protein G beads without antibody (lanes 4 and 7; −,−). Lane “M” is the molecular weight marker.

BKAPs were identified from chick membrane/cytoskeletal and cytoplasmic fractions using BK co-IP, 2-D gel electrophoresis, and LC-MS/MS analysis. This procedure involved the removal of non-specific binding proteins, and isolation of BKAPs with controls using non-immunoprecipitated matrix assays and irrelevant antibody of the same type [9]. Different fractionated protein extracts of chick cochleae were resolved across a broad range of pH and weights by 2D gel electrophoresis for both immunoprecipitated and non-immunoprecipitated gels. BK-immunoprecipitated 2D gels had a high gel-to-gel reproducibility with a total of 73 discrete spots from membrane/cytoskeleton fractions and 52 from cytoplasmic fractions (Figure 1A, 1B). Non-immunoprecipitated gels, which reflected the total cochlear proteome, showed a total of 253 and 196 features from membrane/cytoskeletal and cytoplasmic fractions, respectively, including minor variants (Figure S1A, S1B). Comparisons of total proteome with BK-immunoprecipitated gels revealed that nearly 32.4% and 29% of the protein spots were contributed from membrane/cytoskeletal and cytoplasmic fractions, respectively. In contrast, 2-D gel electrophoresis of matrix and antibody specificity controls showed no protein spots (Figure S1C–F), inferring that our immunoprecipitation assay had few if any false positive proteins.

Our approach to identifying proteins with high confidence from their peptide matches was defined in two steps: 1) proteins identified by MS had a molecular weight variance of ±5 kDa between observed (gel) and theoretical (MS) and 2) protein ID was dependent on a minimum of four peptide sequences with MASCOT score values greater than 150 [18]. Consequently, from the original 663 unique proteins assessed by Scaffold, there remained a total of 60 and 50 distinct proteins from membrane/cytoskeletal and cytoplasmic fractions, respectively. These final numbers excluded protein duplication, of which ∼10 proteins were common to both fractions and included actin cytoplasmic-2, γ-enolase, and HSP70, among others. From these 110 proteins, 91 (82.7%) had ≥7 peptides and 99 (94.3%) had a MASCOT score ≥200 with ≥7% sequence coverage (Table S2A, S2B). A comparison of the chick 2D-gel profile with that of the previously published mouse profile [9] revealed an overlapping, but not identical pattern. Thirty-seven percent of the BKAPs are common to both chick and mouse, while the remaining 63% are novel to chick. These results further confirm that our fractionated 2D-gel proteome profile did not identify any protein G Sepharose sticky proteins. The Scaffold reverse database approach was used to determine the false-positive discovery rate, which was <0.5% for all sequence collections. The assigned sequences had both *b* and *y* ions present in the tandem mass spectra and there were≤three modifications on the peptide. Moreover, none of the proteins identified in the reverse database achieved the MASCOT cutoff score set as a positive ID.

To corroborate the results obtained by the LC-MS/MS approach, we selected 16 representative examples of BKAPs and confirmed their BK association by reciprocal coIP (Figure 1C), using commercially available antibodies. Some of these proteins associate with BK in other systems, whereas others are new associations. All BKAPs were able to coIP a protein that was comparable to an IP of BK at ∼90 or 120 kDa, depending on the splice variant [19], while pre-adsorption controls were clean.

### Biological properties and functional composition of BK interaction networks

Protein-protein interaction networks complemented our original proteomic data. Secondary interactors with primary BKAPs were identified using the IntAct [13] database, since evidence of multiple interactions, as found in IntAct, support high confidence interaction [20]. To accomplish these analyses, murine (*Mus musculus*) interologs in the database were used, due to a lack of available chick interaction data. Interolog mapping, that is, the transfer of interaction annotation from one organism to another using comparative genomics, is an accepted technique in cases where there is limited or no data on the organism of interest, enabling the expansion of an interaction network. While it is accepted that these secondary interactors can only be regarded as predictions, there is good evidence that protein–protein interactions can be transferred when a pair of proteins has a joint sequence identity >80% [21]. Therefore, it is reasonable to expect that the majority of these interactions will be conserved between mouse and chicken.

**Figure 2:**
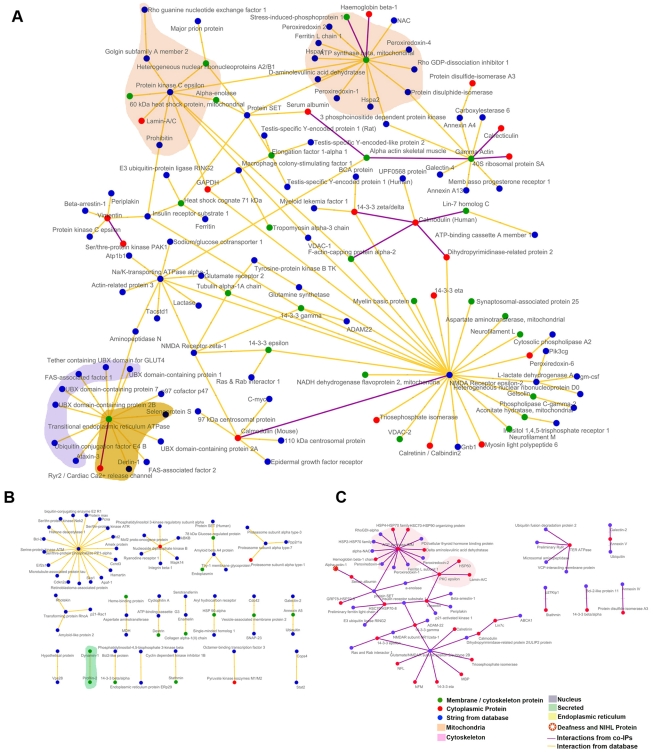
The BK interactome of the cochlea. (**A**) Visualization of primary and secondary BKAPs using Cytoscape revealed 19 networks involving 193 proteins and 190 interactions. Of these proteins, 119 are nodes linked with 136 edges to form a single global network. Within this network are 8 major hubs consisting of a single node connected to 6 or more nodes that may or may not be linked to the larger network. The central nodes in these major hubs include protein kinase C, ATP synthase β, γ-actin, protein SET, Na/K- transporting ATPase, transitional ER ATPase (VCP), calmodulin, and NMDA receptor. (**B**) The remaining BKAPs consist of 71 nodes and 53 edges that form 18 smaller, distinct modules with 19 or fewer nodes. There are two hubs consisting of a central node with 6 or more proteins. The central nodes consist of ser/thr phosphatase PP1α and nucleoside diphosphate kinase B. (**C**) The interactome containing BKAPs common to both mouse and chick consists of a single global network with 45 nodes linked with 47 edges. Within this network are 2 major hubs consisting of a single node connected to more than 6 nodes that may or may not be linked to the larger network. Central nodes in these hubs include NMDA receptor, and ATP synthase. The remaining BKAPs consist of 14 nodes and 9 edges that form 5 smaller modules with 5 or fewer nodes. Different-colored nodes represent contributions from either membrane/cytoskeletal or cytoplasmic fractions, or from the IntAct database. Different-colored edges indicate interactions derived from the BK coIP assays or from IntAct. Colored fields represent portions of the network that are located in different subcellular locations. BKAPs involved in deafness/NIHL are indicated by a star symbol.

This search resulted in a total of 193 proteins involved in 190 binary interactions, including complementary A-B and B-A interactions, when limiting the search to only those interactions classified as physical, thus, excluding cosedimentation (i.e., colocalization) data (Table S3). Visualization with Cytoscape [14] shows that the majority of proteins (61.5%) is linked and forms one large interaction network (Figure 2A). The remaining 38.5% are dispersed among 18 smaller networks with fewer than 19 nodes (Figure 2B). The larger global network has 8 major hubs, containing a central protein connected to six or more partners, some of which are linked to the larger global network. Four of these tightly linked hubs can have a role in apoptosis, including transitional ER (TER) ATPase (aka VCP), ATP synthase β protein SET, and protein kinase Cε [22]–[25]. Three major hubs contribute to neuronal function and include serine/threonine protein phosphatase 1α, NMDA receptor, and nucleoside diphosphate kinase [26]–[28], while the remaining hubs, Na/K-transporting ATPase α-1 and calmodulin [29], [30] are involved in metabolic processes and Ca^2+^ binding, respectively (Figure 2B, 2C). Nearly 23% of these binary interactions are common to both chick and mouse and contain two major hubs, ATP synthase and NMDAR (Figure 2C).

**Figure 3:**
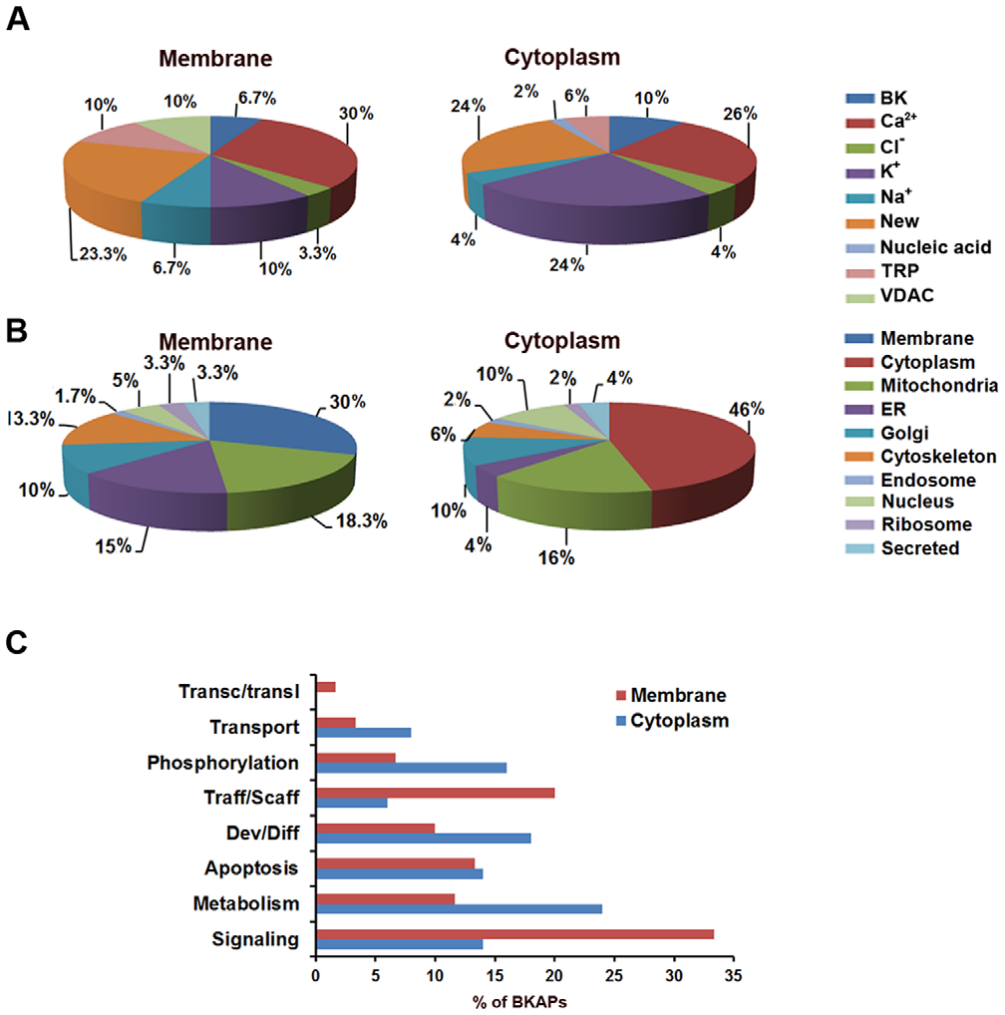
BKAP relationships to ion channels, subcellular localization, and cellular process. (**A**) The manual mining of PubMed revealed ion channel partners for BKAPs isolated from membrane and cytoplasmic fractions of chick cochlea. Two major partners are Ca^2+^ and K^+^, whereas ∼24% are new associations and 9–10% were reported previously as BKAPs. Fewer than 10% are found as partners of channels such as Na^+^, TRP, Cl^−^, and VDAC. (**B**) A search of UniProtKB found BKAPs localized in different subcellular compartments. While a majority are found in the membrane, cytoplasm and mitochondrion, >10% are localized to the ER, Golgi, and cytoskeleton. (**C**) Mining of Gene Ontology, GOSlim, and PubMed revealed that >15% are relegated to cellular processes that include signaling, metabolism, development/differentiation, trafficking/scaffolding, and phosphorylation.

BKAPs were classified according to prior ion channel associations, subcellular localization, and cellular processes (Figure 3A–C; Table S4). To better determine BKAP involvement with other ion channels, we manually mined curated literature annotations (Figure 3A). These data reveal that for both membrane/cytoskeletal and cytoplasmic fractions, ∼76% of BKAPs have a prior association with various types of ion channels, whereas the remaining 24% are novel protein-ion channel interactions. These data do not reflect the previous interactions reported in mouse [9]. The majority of BKAP interactions are with Ca^2+^ (30%, 26%) and K^+^ (10%, 24%) channels. Remaining interactions for each respective membrane vs. cytoplasmic fractions are in descending order, BK (6.7%, 10%), TRP (10%, 6%), Na^+^ (6.7%, 4%), voltage-dependent anion channel (VDAC) (10%, 0%), Cl^−^ (3.3%, 4%), and nucleic acid (0%, 2%).

Primary BKAPs were consigned to a subcellular compartment based on information from Swiss-Prot and GoPubMed databases (Figure 3B; Table S4). The majority are membrane (30%) and cytoplasmic (46%) proteins, whereas 18.3% and 16% are localized to the mitochondrial membrane and matrix, respectively. The second major compartment determined for each fraction was the Golgi (10%, 10%), followed by proteins related to the ER (15%, 4%), cytoskeleton and microtubules (13.3%, 6%), and nucleus (5%, 10%). The fewest number of BKAPS were secretion- (3.3%, 4%), ribosomal- (3.3%, 2%), and endosomal-related (1.7%, 2%). These results support our previous findings [9], especially in regard to mitochondrial associations, suggesting evolutionary conservation in avia and mammals.

BKAPs were classified according to their cellular processes by manual data mining of PubMed literature annotations and Gene Ontology databases (Figure 3C, Table S4). BKAPs were associated with eight specific cell processes. From both membrane/cytoskeletal and cytoplasmic fractions, a majority of proteins were involved in signal transduction- (33.3%, 14%) and metabolism- (11.7%, 24%) related processes, while remaining proteins were consigned to development/differentiation (10%, 18%), apoptosis (13.3%, 14%) trafficking/scaffolding (20%, 6%), phosphorylation (6.7%, 16%), transport (3.3%, 8%), and transcription/translation (1.7%, 0%).

### Conservation of interactions

**Figure 4:**
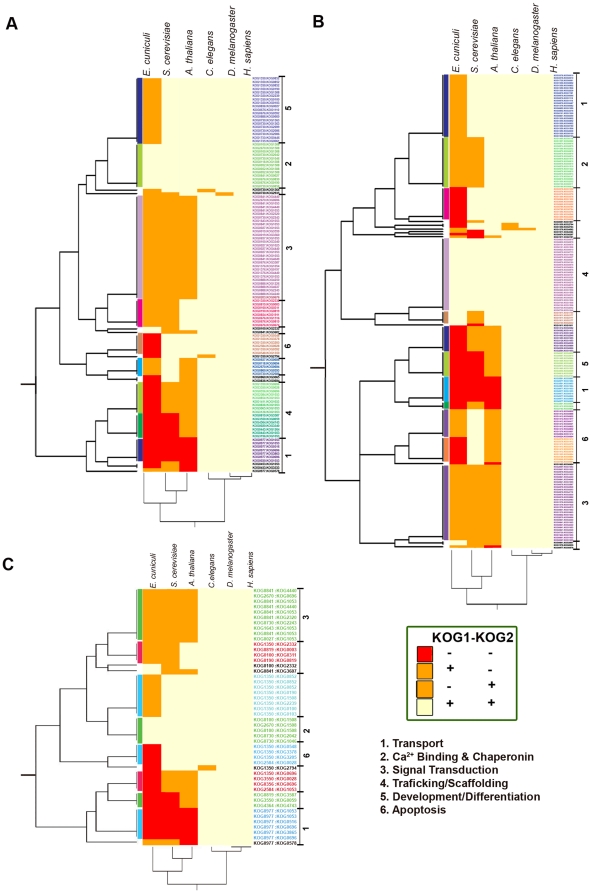
Phylogenetically conserved patterns of iKOGs across six species. Dendrograms are shown for interologs common to (**A**) chick only, (**B**) mouse only, or (**C**) both chick and mouse. Each row in a plot corresponds to one of six eukaryotes and each column corresponds to the iKOG pairs. Both (+,+), neither (−,−) or either (+,− or −,+) member of an iKOG pair is conserved for each eukaryote. Some profiles are separated into conserved functional clusters such as transport, Ca^2+^ binding and chaperonin, signal transduction, etc.

To further determine the conservation of primary/secondary BK interactions, we constructed a phylogenetic profile using data from chick and the previously published dataset for mouse [9] (Figure 4A–C). The binary interaction datasets from an IntAct analysis of chick (199) and mouse (256) totals 455, of which we were able to assign each member of 303 interacting pairs a KOG_ID, available via the STRING database. Of the 303 interacting pairs, 48 are common to both chick and mouse, whereas 66 and 141 are present in chick and mouse, respectively.

A phylogenetic profile of iCOGs was generated, using the six different species available at the NCBI KOG, to determine which binary interactions are conserved in both chcicken, mouse, and their interologs (Figure 4; Table S5A–C). Results of a matrix cluster analysis, plotted as dendrograms, showed that one or both KOGs of a pair was primarily absent in non-vertebrates such as *E. cuniculi*, *S. cerevisiae*, and *A. thaliana*. Additionally, these plots show distinctly conserved clusters of iKOGs with a common function, numbered 1–6; clusters of protein pairs with disparate functions are not numbered. The most conserved functional iKOGs across all six species are 2 (chick only) and 4 (mouse only), which are composed of Ca^2+^-binding/chaperonins and trafficking/scaffolding proteins, respectively (Figure 4A–C). Moreover, cluster 2 shares protein pairs common to both chick and mouse, while cluster 4 is composed of different pairs of proteins for both of these species (Figure 4A–C). The second most functionally conserved clusters of iKOGs are 5 and 6 (chick) and 1 (mouse) (Figure 4B, 4C). These clusters contain transport, development/differentiation, and apoptotic proteins, respectively, and are phylogenetically conserved across 5 species. Moreover, clusters 1 and 6 contain functional proteins common to both chick and mouse. Signal transduction proteins comprise most of functional cluster 3, which is common to both mouse and chick as well as to *C. elegans*, *D. melanogaster*, and *H. sapiens* (Figure 4A). These analyses demonstrate the biological relevance of these interactions, since many are conserved phylogenetically and define essential sets of protein activities in relation to the BK channel.

### The BK interactome reflects cell life/death events

**Figure 5:**
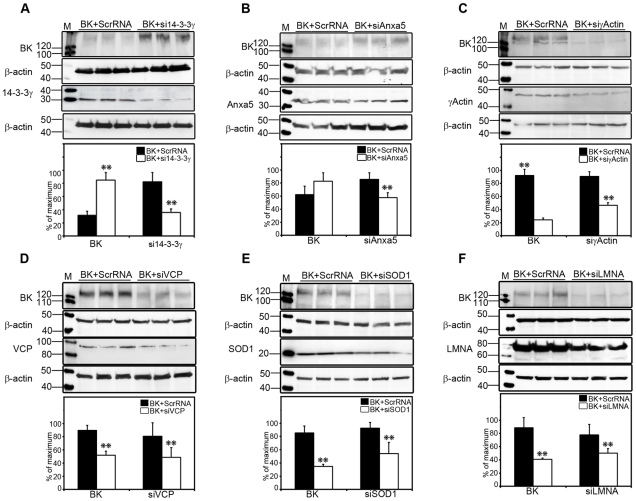
Characterization *in vitro* of BK and BKAPs using siRNA. (**A,B**) Transfection of CHO cells with HA-BK and siRNAs reveals that a knockdown of endogenous 14-3-3γ and AnxA5, increases BK expression compared to scRNA controls. (**C–F**) Silencing γ-actin, VCP, SOD1, and LMNA reduces BK expression compared to scRNA controls. Densitometry measurements were normalized to the highest densitometric value (normalized to 100%) within a given set of lanes, consisting of triplicates for sc- and siRNA-treated cells. Statistical significance was determined using an unpaired, two-tailed *t*-test to obtain **, *p*<0.001. Error bars represent the S.D.

An HA-tagged BK-DEC variant, cloned from cochlear tissues, was designed to determine the effect of selected BKAPs on BK expression. CHO cells were transfected with this variant and selected BKAPs knocked down using RNAi. Six BKAPS were chosen, representing different aspects of cellular processes and location such as survival (14-3-3), trafficking (Anxa5), cytoskeletal (γ-actin), nucleus/structural (LMN), mitochondrial (SOD), and proteasomal (VCP). HA-BK was measured in response to BKAP silencing (Figure 5A–F). Using siRNAs, a knockdown of endogenous 14-3-3γ (33%) and AnxA5 (23%) resulted in a respective 25% and 17%, increase in HA-BK expression, compared to scRNA controls. Silencing of γ-actin (21%), LMN (19%), VCP (32%), and SOD1 (38%) caused BK to decrease by 31%, 48%, 38%, and 51%, respectively.

**Figure 6:**
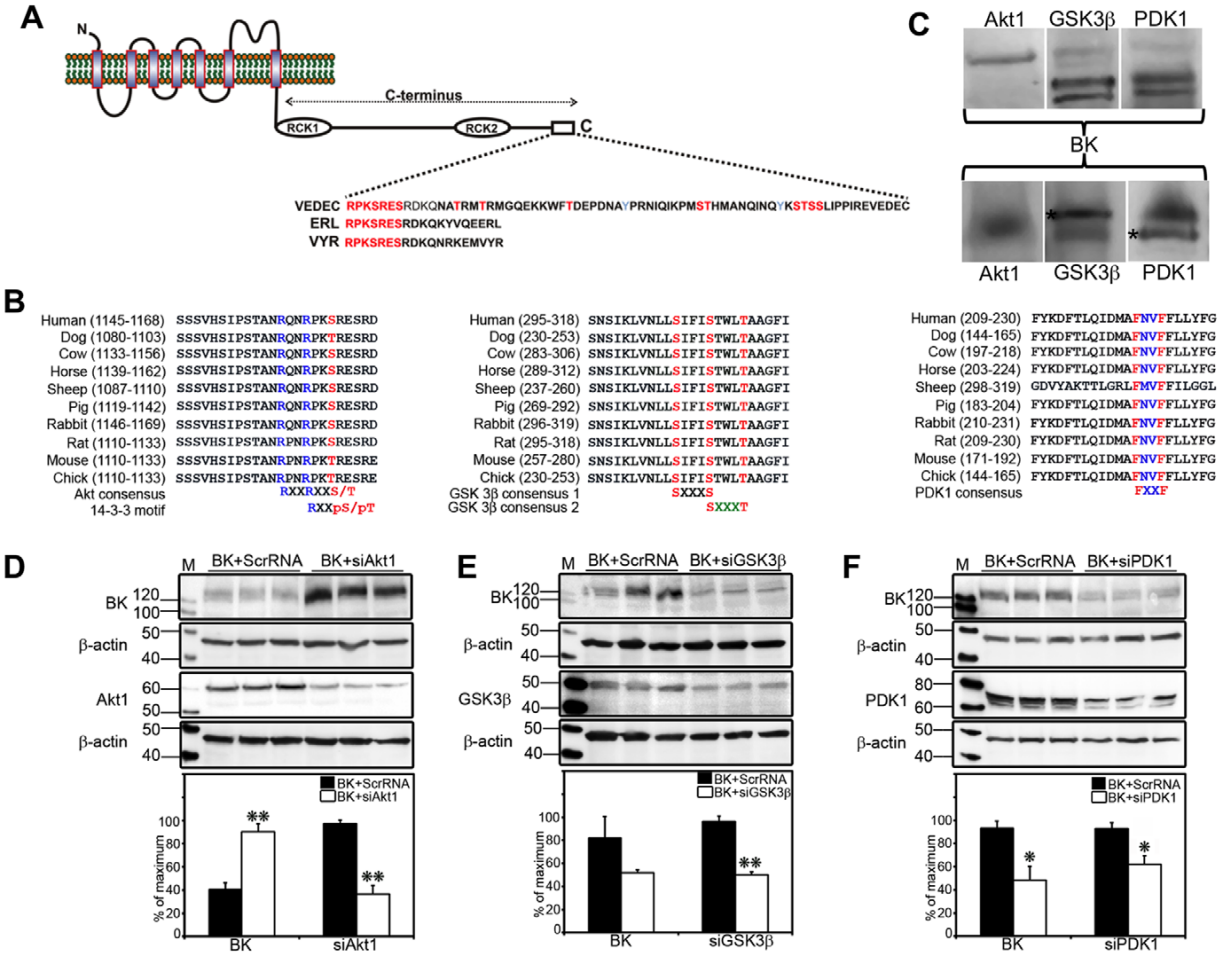
Putative binding sites for life/death signals alter BK expression. (**A,B**) The BK C-terminus of 10 different vertebrates has putative binding sites for Akt1, 14-3-3γ, GSK3β, and PDK1. (**C**) Akt1, PDK, and GSK3β interact with BK, as verified by reciprocal coIP using total cochlear lysate. Label above photos indicate antibody used for coIP, whereas the label below photos indicate the protein coimmunoprecipitated. Akt1 coimmunoprecipitated one isoform of BK (130 kDa), whereas GSK3β and PDK1 coimmunoprecipitated multiple isoforms (90–130 kDa). In the reciprocal experiment, BK coimmunoprecipitated Akt1 (56 kDa), GSK3β (47 kDa), and PDK1 (63 kDa). The IgG band is signified by an (*). (**D–F**) CHO cells were transfected with HA-BK and scRNA, or HA-BK and siRNAs for Akt1, PDK, and GSK3β. HA-BK increased with knockdown of Akt and decreased with knockdowns of GSK3β and PDK1. Densitometry measurements were normalized and statistical significance determined in D–F as in Figure 5 to obtain *, *p*<0.01, **, *p*<0.001. Error bars represent the S.D.

BK sensitivity to Ca^2+^ can be regulated through channel phosphorylation by serine-threonine and tyrosine kinases [5]. Thirty putative phosphorylation sites were identified from seven different BK splice variants [4]. Our BK-DEC variant has an additional 60 amino acids, containing 11 serine/threonine and tyrosine residues at the extreme end of the C-terminus (Figure 6A). The effects on BK in relation to the six different BKAPs led us to examine an important survival hub protein, the serine/threonine kinase, Akt/PKB (reviews in [31]). This line of reasoning was underscored by previous evidence showing that BKAPs, such as 14-3-3, LMN, and VCP interact or are part of the Akt pathway [31]–[33]. Sequence analysis of BK revealed that aa 1122–1128 contain an RxxRxxS/T motif (Figure 6B), which is a potential Akt1 substrate binding site [31]. This motif is conserved in BK mouse and rat orthologs, and can vary between aa 1079 and 1169 in other mammalian species. Akt1 substrate binding sites are not found in reptiles, amphibians and fishes, suggesting that this motif is relegated to mammals. To further examine the possibility that BK may associate with other pro-/anti-apoptotic kinases, the BK-DEC sequence was examined for motifs binding 14-3-3, GSK, and PDK, the latter two of which can regulate Akt effects. Depending on the species, sequence analyses showed a 14-3-3 binding site (RXXS/T) between aa 1091–1164, a GSK3β site (SXXS) between aa 239–314, and a PDK1 site (FXXF) between aa 156–314 (Figure 6B).

We determined whether the BK/Akt effect was a result of direct interactions between these proteins, given these putative binding sites (Figure 6C). Reciprocal coIPs revealed that BK coimmunoprecipitated Akt (56 kDa) and vice-versa, suggesting a direct physical interaction. Similar interactions were revealed for GSK3β (47 kDa) and PDK1 (63 kDa) using reciprocal coIPs. We then tested whether silencing Akt, GSK3β, and PDK1 affects BK expression (Figure 6D–F). The result was a 57% increase in BK expression with a 54% decrease in Akt1, whereas BK decreased 45% and 30% with a decrease in PDK1 (31%) and GSK3β (46%) (p<0.05), respectively. The loading control, β-actin, showed no differences between sc- and siRNA-treated conditions (p>0.05).

## Discussion

We have taken a systems-biology approach using coIP with 2-D gels, mass spectrometry, and bioinformatics to identify BK interactions that contribute to cochlear metabolism, signal transduction, phosphorylation, and apoptosis. The limitations of coIP-based assays and mass spectrometry include false positive and false negative discovery rates. Therefore, when we set out to develop the BK interaction network, we implemented several strategies to reduce these limitations. For example, non-specific antibody and bead controls, Scaffold analysis against a reversed database, high peptide coverage (minimum of four) for protein ID, and high MASCOT score cut-off values minimize the likelihood of false positive interactions [34], [18]. The guidelines of these parameters produced a high-confidence BK interactome.

### Conservation of interactions across species

A phylogenetic profile for interacting protein families was constructed using the eukaryotic (orthologous group) ortholog database. The BK interaction network contains protein interactions that are conserved from microsporidia to mammals, including conservation between chicken and mouse. This result suggests that certain parts of these interactomes have evolved through the preferential addition of interactions between lineage-specific proteins [35]. Clusters 2 and 4, which were highly enriched for Ca^2+^-binding and trafficking/scaffolding BKAPs, respectively, were among the most highly conserved. Common Ca^2+^-binding proteins were found across all six species. These protein families appeared very early in the history of cells to maintain extra- and intracellular Ca^2+^ homeostasis, mediating Ca^2+^-induced cell damage and maintaining cell survival [36]. The trafficking/scaffolding iKOGs of mouse were found to have common ancestors across all six species examined, whereas those of the chick were common only to three. This outcome suggests a possible divergence between chick and mouse with regard to trafficking/scaffolding protein-protein interactions. Numerous trafficking/scaffolding proteins evolved to dominate the mode of multiple protein interactions found in a complex assembly at the plasmalemma and excitatory synapses [37]. Further investigations of, for example, their domains may provide insights into underlying interactome evolution, as evidence suggests domains can take evolutionary jumps associated with changes in function [37].

Two primary hub proteins shared by both chick and mouse are NMDA2B receptors and the enzyme, ATPase, which is found in the inner mitochondrial membrane. These hubs connect 45 proteins in a conserved network of primary and secondary interactions. The NMDA receptor, which acts as a secondary partner, colocalizes with BK, as they interact via Ca^2+^ influx through NMDARs, which activates the BK channel [38]. Presently, there is no evidence for a direct physical interaction, nor is there one suggested by the interactome. NMDA2B receptors are present in spiral ganglion cells and adjacent to inner and outer hair cells of human cochlea [39]. Similarly, BK channels are found in ganglion cells and at the base of outer hair cells [19].

ATP synthase, an enzyme that converts ADP to ATP using inorganic phosphate, is found on the inner mitochondrial membrane, as is the BK channel (reviews in [40]). Presently, there is no evidence for a direct physical interaction, although the interactome described herein suggests a relationship. ATPase may be part of a larger multiprotein complex that forms the permeability transition pore (PTP), which is linked to apoptosis that commences with Ca^2+^ overload. Both BK_mito_ and K_ATP_ channels are thought to protect against ischemia by inhibiting the PTP, either by direct physical interaction or by inhibiting the factors that initiate the opening of the PTP [40]. Recent evidence suggests the presence of BK_mito_ in the cochlea [9], [19], although its relation to ATP synthase and the PTP requires further studies.

### Silencing of apoptotic-related BKAPs alters BK expression

BKAPs chosen from the interactome have functions related to pro- or anti-apoptotic events. Knockdown of VCP, LMNA, SOD1, and γ-actin decreased BK expression. VCP is a hub in the BK interactome and a member of the ATPase-associated (AAA) family that binds ubiquitin and acts as a chaperone of proteins marked for degradation in the proteasome. Similar to nucleoside diphosphate kinase (NDK), another BK hub protein, VCP has a role in ER stress-induced apoptotic execution [41]. While NDK was found outside the large interactome, previous evidence suggests that NDK participates in this network by virtue of the ER-associated SET complex found in the large network. Protein SET is an inhibitor of NDK/NM-23 [42] and when removed allows cell death via DNA degradation [43]. Consequently, when considered together, these interactions generate at least 18 BKAPs potentially involved in apoptosis, including ubiquitin regulatory X (UBX) -containing proteins and Fas-associated Factor (FAF). Interestingly, NDK increases the activity of a Ca^2+^-activated K^+^ channel (KCa3.1) in CD4 cells, by directly binding and phosphorylating histidine 358 at the C-terminus [44]. Here, as in vascular smooth muscle, a Ca^2+^-activated K^+^ channel regulates Ca^2+^ influx. In the inner hair cells of mammals, BK channels are found in extrasynaptic regions, at the apex of the cell, and near the sites of Ca^2+^ influx via transduction channels in the stereocilia. Thus, the regulation of Ca^2+^ as well as K^+^ via these channels might have a role in not only signal processing but also cellular homeostasis.

BK decreased with the silencing of LMNA, SOD1, and γ-actin in CHO cells. All three are important to cell viability since they are antiapoptotic. The nuclear lamina, a network of lamins and membrane-associated proteins, is attached to the inner face of the nuclear membrane. Lamins are among the first apoptotic targeted proteins during the breakdown of the nuclear structure [45]. In contrast, SOD1 resides both in the cytosol and mitochondria and is an antioxidant isoenzyme that dismutates superoxide anions to H_2_O_2_ in response to oxidative stress. Its mimetic, tempol, can activate BK channels [46]. Future studies are needed to examine these relationships, since BK may reside in the nuclear envelope [47] and/or nucleus [48], [19] as well as in the mitochondria of cochlear cells [9], [19]. Finally, as with LMNA and SOD1, actins play a role during apoptosis, since they are targeted by caspases, leading to their disassembly [49]. BK associates with both α- and γ-actin, which with leptin can cluster BK channels at synapses [50], [51].

In contrast to the previous four proteins, silencing of Annxa5 and 14-3-3 increased BK expression. Trafficking and cytoskeletal proteins, such as annexin and actin, work in concert to maintain membrane complexes regulated by Ca^2+^, which alters the conformation of Annxa5 [52]. One of these complexes consists of apolipoprotein A1 (ApoA1) and annexin, which form in a Ca^2+^-dependent manner [53] and of which apolipoprotein, found in cochlear hair cells, alters the biophysical characteristics of BK [54]. In comparison, 14-3-3 reportedly interacts with dSlo via the accessory subunit dSlob in *Drosophila* [5]. Our data, however, suggest a direct relationship with a putative binding site for 14-3-3. A template for this interaction is the TASK K^+^ channel/14-3-3 relationship, where the C-terminus of TASK directly interacts with multiple isoforms of 14-3-3. However, unlike the TASK/14-3-3 interaction, where 14-3-3 increases TASK cell surface expression [55], 14-3-3 decreases BK. These differences may hinge on other BKAP kinases, since 14-3-3 interacts with kinases such as GSK [56]. Interestingly, GSK activation via phosphorylation increases cell viability, but is moreover correlated with the coupling of GSK to 14-3-3 [56]. How these kinases might affect each other and their interactions with BK may be related to a kinase central to survival, Akt.

### Life/death signals alter BK expression

The BK channel has a long C-terminus with a number of phosphorylation sites [4]. Primary and secondary partners of BK revealed apoptosis-related signals, leading us to examine kinase effectors that may bind to this channel. Central to these life/death effectors is the serine/threonine kinase Akt. Akt has a central role in cell survival, inhibiting apoptosis by phosphorylating and inactivating targets such as BAD [31], and caspase-9 [31]. Moreover, Akt binds to phosphatidylinositol (3,4,5)-trisphosphate at the plasma membrane, initiating activation by phospholipid binding and phosphorylation and using 14-3-3 as a substrate. [31]. Previous studies suggest Akt regulates BK activity as part of an estrogen transduction-signaling component, where estrogen stimulates nNOS via the PI3 kinase/Akt pathway [57]. Akt can also mediate plasmalemma expression of BK through neuregulin [58]. However, our data suggest direct Akt/BK interaction via Akt and 14-3-3 binding sites that overlap at the C-terminus of BK. Knockdown of Akt1 and 14-3-3 increases BK expression, suggesting that BK is kept in check by these kinases. In contrast, GSK3β and PDK1 knockdown resulted in decreased expression of BK in CHO cells. Interestingly, Akt inhibits GSKβ, an initiator of apoptosis whereas PDK1 partially activates Akt via phosphorylation [31]. These results, when taken in concert with putative binding sites for GSK3β, PDK, 14-3-3, and Akt implicate the phosphorylation of BK in cell death/survival. Moreover, they beg the question of whether these kinases act separately or in concert to activate/deactivate BK directly and whether their relationship to their counterparts in the larger cell death/survival pathways regulates cell viability via BK?

The outcome of our study reveals a highly connected protein network that forms several functional regulatory pathways whose interactions are conserved across a number of eukaryotes. Intriguingly, we found putative BKAPs that are involved in both BK phosphorylation, and cell survival/apoptosis. Thus, the BK channel may have a role not only in excitation, but possibly cellular homeostasis, as a function of possessing motifs that bind death/survival kinases. BK channel participation in saving cells from ischemia is well documented in relation to its role in mitochondria, as is the regulation of cellular homeostasis by K^+^ efflux through channels in the plasmalemma (reviews in [59]). Given these findings, the importance of BK in hair cells under stress, as would occur during noise-induced hearing loss, cannot be underestimated, especially in light of studies that show BK knockouts are protected from such a loss [8]. Our data provide a foundation for future studies of an expanded network and its relation to where and when interactions take place, how they are regulated, and the logic of this complex biological network in relation to hearing.

### Data Availability

The interactions in this study have been submitted to the IMEx consortium (http://imex.sourceforge.net) through the IntAct database (http://www.ebi.ac.uk/intact/, accession number IM-9475).

## Supporting Information

Figure S1 **Images of 2-D gel electrophoresis of chick cochlear proteome and controls.** Two-dimensional gel electrophoresis of the total proteome for the (**A**) membrane/cytoskeletal and (**B**) cytoplasmic fractions shows 253 and 196 visible features, respectively. (**C**) Results for membrane/cytoskeletal and (**D**) cytoplasmic fractions of mouse cochleae incubated with protein G beads without anti-BK antibody. The non-specific proteins were washed and eluted from the beads as in the experimental groups. (**E,F**) Fractions, as before, were incubated with beads bound with a non- specific antibody to the cochlea, anti-VSV-G antibody. Any non-specific proteins captured were washed, eluted, and analyzed, as described previously.
(TIF)

Table S1 List of antibodies and primers used in the study.
(XLS)

Table S2 List of BKAPs identified by LC-MS/MS from the membrane/cytoskeletal and cytoplasmic fractions.
(XLS)

Table S3 List of BKAPs from membrane and cytoplasmic fractions and their interactions with binary partners, as determined by IntAct. These proteins appear in the interactome.
(XLS)

Table S4 Primary BKAPs, from membrane/cytoskeletal and cytoplasmic fractions, categorized according to organelle location, ion channel association, and cellular process.
(XLS)

Table S5 UniProt IDs of primary and secondary partners with their corresponding KOG_IDs common to mouse alone, chick alone, and mouse and chick together.
(XLS)
