# Correction: Conserved BK Channel-Protein Interactions Reveal Signals Relevant to Cell Death and Survival

**DOI:** 10.1371/annotation/15e95626-9f5c-4882-ba78-826b80c48028

**Published:** 2012-01-27

**Authors:** Bernd Sokolowski, Sandra Orchard, Margaret Harvey, Settu Sridhar, Yoshihisa Sakai

There was an error in the accession number in the Data Availability section of the Discussion. The correct accession number is IM-15154. 

