# Supplementary figures and images for: Conserved BK Channel-Protein Interactions Reveal Signals Relevant to Cell Death and Survival
Source: PLoS One. 2011 Dec 9;6(12):e28532. doi: 10.1371/journal.pone.0028532 (PMC3235137; doi:10.1371/journal.pone.0028532)

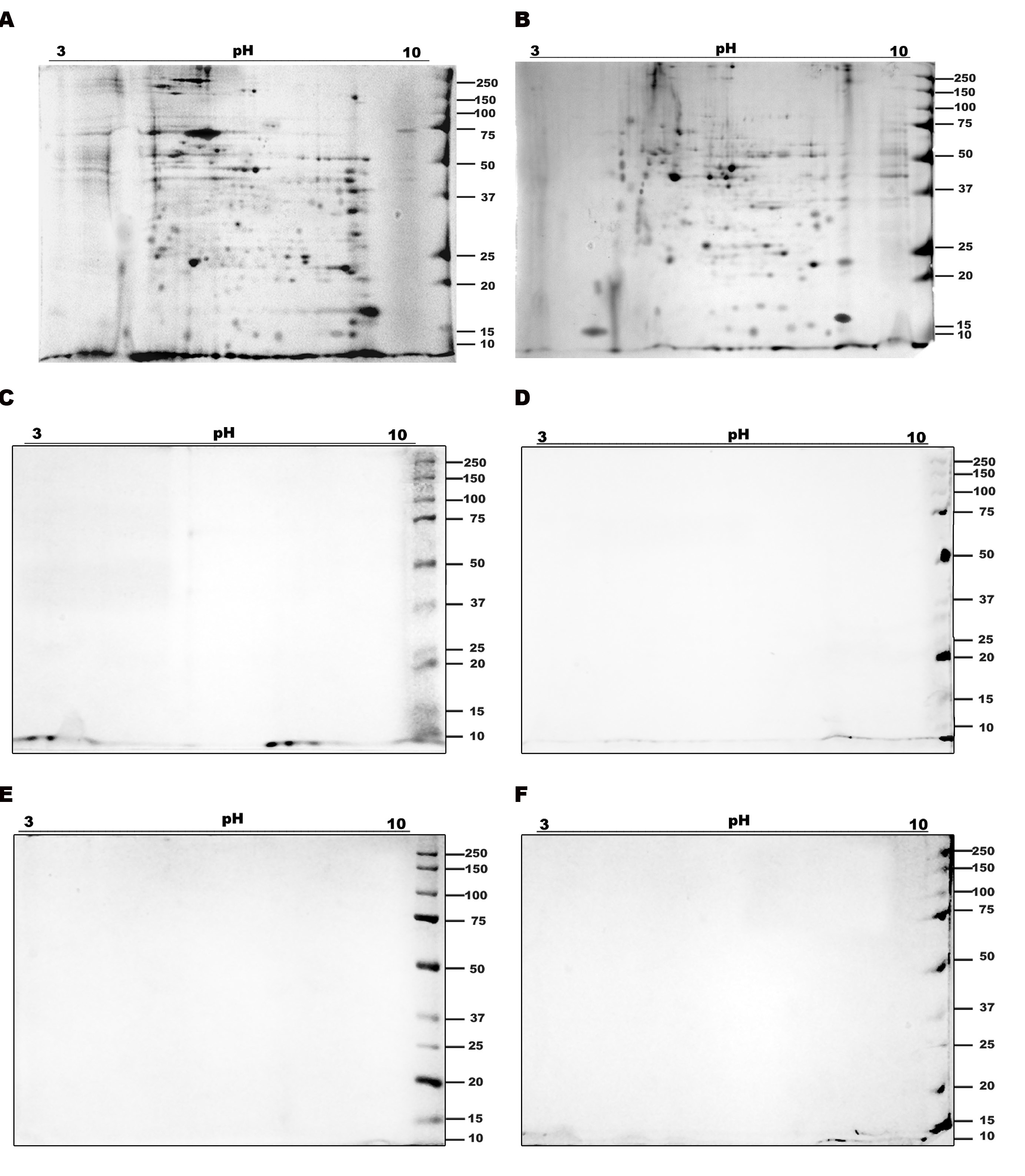

Supplement: Figure S1 — Images of 2-D gel electrophoresis of chick cochlear proteome and controls. Two-dimensional gel electrophoresis of the total proteome for the (A) membrane/cytoskeletal and (B) cytoplasmic fractions shows 253 and 196 visible features, respectively. (C) Results for membrane/cytoskeletal and (D) cytoplasmic fractions of mouse cochleae incubated with protein G beads without anti-BK antibody. The non-specific proteins were washed and eluted from the beads as in the experimental groups. (E,F) Fractions, as before, were incubated with beads bound with a non- specific antibody to the cochlea, anti-VSV-G antibody. Any non-specific proteins captured were washed, eluted, and analyzed, as described previously. (TIF) [file pone.0028532.s001.tif]
